# Systematic review of determinants of mortality in high frequency oscillatory ventilation in acute respiratory distress syndrome

**DOI:** 10.1186/cc4824

**Published:** 2006-02-20

**Authors:** Casper W Bollen, Cuno SPM Uiterwaal, Adrianus J van Vught

**Affiliations:** 1 University Medical Centre Utrecht, The Netherlands

## Abstract

**Introduction:**

Mechanical ventilation has been shown to cause lung injury and to have a significant impact on mortality in acute respiratory distress syndrome. Theoretically, high frequency oscillatory ventilation seems an ideal lung protective ventilation mode. This review evaluates determinants of mortality during use of high frequency oscillatory ventilation.

**Methods:**

PubMed was searched for literature reporting randomized trials and cohort studies of high frequency ventilation in adult patients with acute respiratory distress syndrome. Data on mortality and determinants were extracted for patients treated with high frequency oscillatory ventilation. Linear regression analyses were conducted to produce graphical representations of adjusted effects of determinants of mortality.

**Results:**

Cohorts of patients treated with high frequency oscillatory ventilation from two randomized trials and seven observational studies were included. Data from cohorts comparing survivors with non-survivors showed differences in age (42.3 versus 51.2 years), prior time on conventional mechanical ventilation (4.0 versus 6.2 days), APACHE II score (22.4 versus 26.1), pH (7.33 versus 7.26) and oxygenation index (26 versus 34). Each extra day on conventional ventilation was associated with a 20% higher mortality adjusted for age and APACHE II score (relative risk (RR) 1.20, 95% confidence interval (CI) 1.15–1.25). However, this association was confounded by differences in pH (pH adjusted RR 1.03, 95% CI 0.73–1.46). Oxygenation index seemed to have an independent effect on mortality (RR 1.10, 95% CI 0.95–1.28).

**Conclusion:**

Prolonged ventilation on conventional mechanical ventilation prior to high frequency oscillatory ventilation was not related to mortality. Oxygenation index was a determinant of mortality independent of other disease severity markers.

## Introduction

Acute respiratory distress syndrome (ARDS) is a clinical condition that is associated with high mortality [1]. Different lung protective ventilation strategies have had an important impact on mortality in ARDS [2]. These strategies are based on the concept that there is a safe window between atelectasis and overdistension of alveoli and have been developed, therefore, with the aim of recruiting alveoli combined with avoidance of high peak inspiratory pressures and thus overdistension. A striking impact of how ventilation can affect outcome has been demonstrated by comparing high tidal volume with low tidal volume ventilation strategies, resulting in a 8.8% reduction in mortality in the latter [3]. The most extreme form of low tidal volume ventilation is represented by high frequency oscillatory ventilation (HFOV). In HFOV, a continuous distending airway pressure is applied upon which pressure waves are produced, with frequencies typically ranging from 5 to 10 Hz. To produce these pressure waves, a HFOV ventilator is equipped with a piston driven diaphragm. A power control regulates the force and distance with which the piston moves from baseline. The degree of deflection of the piston (amplitude) determines the tidal volume [4]. This results in extremely small tidal volumes and, therefore, theoretically, in avoidance of overdistension; at the same time, application of continuous distending pressure prevents atelectasis. Thus, theoretically, these attributes make HFOV an ideal candidate for ventilation of patients with a severe lung disease like ARDS [5,6].

Due to technical restrictions, the first HFOV ventilators only had the power to ventilate infants and small children. A population in which HFOV has been extensively investigated consists of premature neonates with idiopathic respiratory distress syndrome. Although numerous randomized trials have been performed, a clinically relevant difference in mortality or pulmonary outcome compared with conventional mechanical ventilation (CV) has not been established [7]. More recent studies looked at the smallest premature infants and strived to minimize time on CV in order to maximize the effect of HFOV compared with CV [8,9]. Yet, it seemed that elective application of HFOV did not influence pulmonary outcome in most premature infants with idiopathic respiratory distress syndrome [10]. Attention has been shifted, therefore, to identifying subgroups of patients that do benefit from HFOV.

In ARDS, only two randomized trials have been performed in adult patients and one in pediatric patients [11-13]. None of these trials were able to show a significant difference in mortality between HFOV and CV. Studies have also been published that investigated determinants of mortality in HFOV treated patients [14,15]. As in studies with premature neonates, selecting the proper subgroup of patients with ARDS for HFOV treatment will be a main issue in trials comparing HFOV with CV [16]. HFOV treated patients in experimental trials and in non-experimental prospective and retrospective cohort studies were evaluated to identify baseline characteristics that predicted mortality and pulmonary outcome in patients who were selected for HFOV treatment.

## Materials and methods

A literature search was carried out to identify all randomized trials of HFOV performed in adult patients with ARDS. Reports of prospective and retrospective cohort studies were separately collected using the terms 'high frequency oscillatory ventilation', 'acute respiratory distress syndrome' and 'mortality' in PubMed and the Cochrane database. This search was updated until September 2005 with no further time limits. Literature lists of meta-analyses and articles were searched for additional studies. To be included, prospective or retrospective studies had to report on well defined cohorts of patients included over a fixed period of time and address mortality as outcome. Case reports, case series, letters and narrative reviews were excluded. Studies were evaluated regarding selection bias and loss-to follow up by CB.

Data extracted for HFOV treated patients in clinical trials and cohort studies were clinically relevant outcome measures, mortality incidence at 30 days in survivors, incidence of still being ventilated at 30 days, and incidence of survival without being ventilated at 30 days. Baseline characteristics of these cohorts that could be associated with mortality were identified. As well as age, sex, and acute physiology and chronic health evaluation (APACHE) II score, the following quantitative variables were extracted from all studies: ratio of partial arterial oxygen pressure (PaO_2_; mmHg) and fraction of inspired oxygen (FiO_2_); time on CV prior to HFOV (days); oxygenation index (OI), which corresponds to FiO_2 _× mean airway pressure (MAP; cmH_2_O) × 100)/paO_2_; blood gas results (pH and pressure of arterial carbon dioxide (PaCO_2_; mmHg)); and ventilatory settings on CV (peak inspiratory pressure, peak end-expiratory pressure, MAP and FiO_2_).

**Figure 1:**
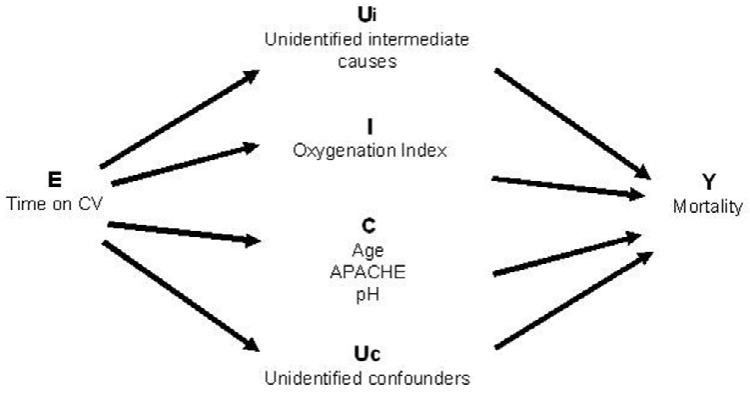
Theoretical causal mechanism of the association between time on conventional mechanical ventilation (CV) prior to initiating high frequency oscillatory ventilation and mortality at 30 days. Conditioning by oxygenation index and age and APACHE II score would block the association if no unidentified intermediate causes or confounders were present. E, exposure; I, intermediate cause; C, confounders; Y, outcome.

Two *a priori *hypotheses were formulated to explain differences in mortality rates between studies in HFOV treated patients: first, a longer duration on CV prior to HFOV causes higher mortality; and second, higher baseline OI is independently associated with higher mortality in HFOV treated patients. These hypotheses have also been raised by others to explain differences between studies [17-19]. However, the association of time on CV prior to HFOV and mortality in HFOV treated patients could be confounded by covariates such as age and disease severity (APACHE II score and pH). In the relationship between time on CV and mortality, OI could be an intermediate cause (Figure 1). Intermediate cause was defined as a factor in a causal pathway; therefore, controlling for an intermediate cause removes the association between an explanatory variable and outcome. If controlling for a well measured intermediate cause does not remove the association, it is not an intermediate cause.

### Statistical analysis

Univariate logistic regression analyses were performed to identify associations between single covariates and binary outcome (for example, survival yes or no). Mean values of reported continuous covariates in survivors and non-survivors in each study were used as covariates. These analyses were weighted by numbers of survivors and non-survivors.

Linear regression analyses were conducted with mortality as dependent outcome and determinants of mortality as independent variables to create graphical presentations of crude and adjusted effects. For the dependent variable, a linear transformation of incidence of death was calculated by taking the natural logarithm of incidence of death divided by incidence of survival. The weight of an individual study was determined by the inverse of the variance of that study.

Multivariable linear regression was used to deal with possible confounding factors of the association between hypothesized causal factors (see Materials and methods) and outcome. Furthermore, we explored in these models whether associations between hypothesized causal factors and outcome could be explained by possibly intermediate factors. To that end we investigated whether inclusion in the model of such intermediate factors would indeed attenuate the association between hypothesized causal factors and outcome, which we will refer to as 'blocking of the effects'.

All analyses were conducted using SPSS 12.0.1 for Windows software (SPSS Inc., Chicago, Illinois, USA).

## Results

Using the search term 'high frequency oscillatory ventilation', 693 articles were found. Limiting the search to studies of adults, only 76 articles were left. Of these 76 articles, 2 were randomized trials and 7 observational cohort studies; 3 of these 9 studies were retrospective studies [14,20,21] and 6 were prospective studies [11,13,15,17,18,22]. Prospective studies contributed 83% of the total weight to our analyses. Nine cohorts of HFOV treated patients from two randomized trials and seven observational trials were included in the regression analyses [11-15,17,18,20-22].

Differentiated data on survivors and non-survivors in HFOV could be extracted from eight studies [11,13-15,17,18,20-22]. Pooled comparison of survivors with non-survivors in the observational studies showed differences in all covariates (Table 1). Crude odds ratios for mortality were calculated for covariates separately. The crude odds ratio for time on CV was 1.38. However, patients that did not survive were also more severely ill (APACHE II score 26 versus 22, pH 7.26 versus 7.33 and OI 34 versus 26).

**Figure 2:**
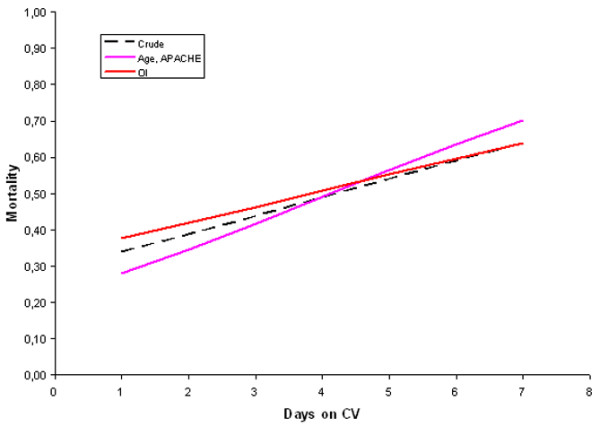
Linear regression analysis of mortality and time on conventional mechanical ventilation (CV). Dashed line, crude analysis; purple line, linear regression adjusted for age and APACHE II score; orange line, linear regression adjusted for oxygenation index.

Coverage of determinants of mortality was complete for age, APACHE II score and OI in seven studies (Table 2). Only five studies supplied both time on CV, pH, PaCO_2 _and OI. The results from weighted multivariate linear regression analyses of mortality incidence in HFOV treated patients are graphically depicted in Figure 2. Adjusting for age and APACHE II score increased the effect of prior time on CV on mortality by 23% per day (relative risk (RR) 1.23 and 95% confidence interval (CI) 1.01–1.49, and RR 1.35 and 95%CI 1.12–1.63 for crude and adjusted, respectively). Addition of OI to the model with age and APACHE II score resulted in a decreased effect of 20% increase in mortality per day on CV (RR 1.20, 95% CI 1.15–1.25).

**Figure 3:**
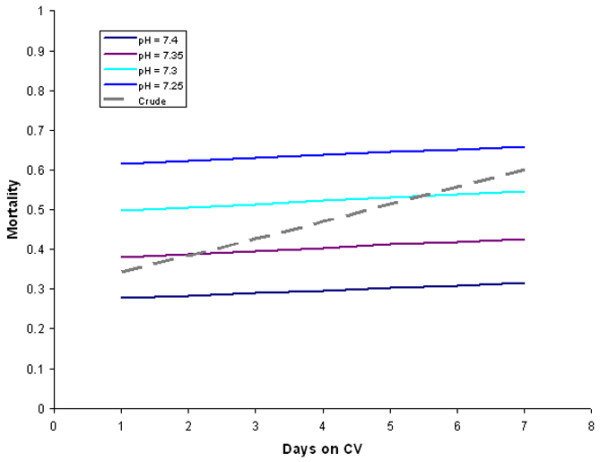
Linear regression of time on conventional mechanical ventilation (CV) on mortality adjusted for different levels of pH. Dashed line, crude analysis; colored lines, linear regression adjusted for pH.

**Figure 4:**
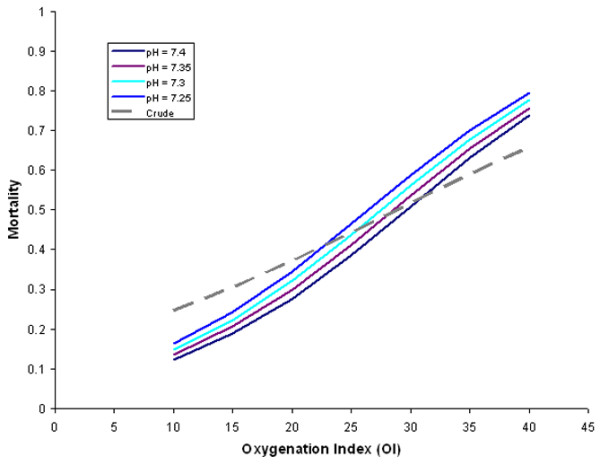
Linear regression of oxygenation index (OI) on Mortality adjusted for different levels of pH. Dashed line, crude analysis; colored lines, linear regression adjusted for pH.

However, the association of time on CV with mortality almost disappeared when adjusting for pH (RR 1.03, 95% CI 0.73–1.46). On the other hand, adjusting for PaCO_2 _did not diminish the effect of time on CV (RR 1.28, 95% CI 1.20–1.36). The association of OI with mortality was less influenced by adjusting for pH (RR 1.10, 95% CI 0.95–1.28). Figures 3 and 4 show the relative contributions to mortality by days on CV prior to HFOV and OI adjusted for different levels of baseline pH. Data on pH could be extracted from only five studies; therefore, a full model with time on CV, age, APACHE II score, pH and OI could not be fitted.

## Discussion

The combined evidence from the randomized trials and observational research of cohorts of HFOV treated patients shows that the association of prior time on CV before initiating HFOV with mortality was confounded by differences in pH between survivors and non-survivors. Furthermore, adjusting prior time on CV by OI as an intermediate cause did not block the effect of prior time on CV. OI, on the other hand, was associated with mortality, independently of age, APACHE II score and pH.

In this review, we combined observational evidence of an additional randomized trial with a previously reported trial and prospective and retrospective cohort studies. *A priori*, two hypotheses that could explain the association between length of ventilation on CV and OI, a marker of pulmonary disease severity, with mortality in HFOV were formulated. Quantitative data were available for two important possible confounders, age and APACHE II score, in seven published cohorts and pH and PaCO_2 _were reported for five cohorts.

Bias inherent to observational research could not be excluded. Selective reporting was not considered to be a major problem, however, because HFOV in adult patients was a relatively new treatment without strong prior beliefs or expectations on the side of the investigators. Missing patients that were treated with HFOV in retrospective analyses was unlikely as well, as this kind of treatment is easily recognized, also in retrospect. Bias due to misclassification and loss to follow up were regarded unlikely in the specific intensive care settings the studies took place. Most determinants consisted of laboratory measurements or ventilatory settings that were not likely to be influenced by observer or recall bias.

There was not enough information to assess possible confounding by other covariates and residual confounding could not be excluded. Furthermore, this meta-analysis was restricted to baseline characteristics. Sequential evolution of determinants over time may be more powerful to predict mortality. However, APACHE II score, pH and OI have been shown to be strongly related to mortality [1]. The OI represents a cost benefit ratio of ventilatory conditions and PaO_2 _yield and is, theoretically, a more sensitive indicator of pulmonary condition than the PaO_2_/FiO_2 _ratio. The inverse relation of mean airway pressure and FiO_2 _with PaO_2 _would render it less susceptible to specific ventilatory settings that were used. Stratified results from the trial by Bollen and colleagues [11] with baseline OI lower or equal to 20, or baseline OI above 20, changed the effect of HFOV on mortality compared with CV. This could indicate that the level of OI determined which patients had the greatest benefit from HFOV.

The association of time on CV with increased mortality adjusted for age and APACHE II score has been reported by several other authors [13,15,17,18]. The proposed mechanism would be through lung damage caused by CV. As we have shown, this hypothesis is not supported by the evidence in our analysis. As we have argued, if the association between time on CV and mortality arises through damage to the lungs caused by CV, we expect that conditioning for OI as a marker of lung injury would explain this association by blocking the effect; that is, by adjusting for OI as an intermediate cause, the association of time on CV with mortality would disappear. However, adjusting for OI did not influence the association between time on CV and mortality. A possibility could be that OI was not an appropriate marker of the intermediate causal pathway and that unidentified intermediate determinants of lung damage remained.

Moreover, the association of prolonged time on CV before initiating HFOV treatment and increased risk of death disappeared by adjusting for pH. It could be argued that pH was an intermediate causal factor. However, adjustment for PaCO_2 _did not influence the association with time on CV and mortality, suggesting that respiratory acidosis due to worsening pulmonary function caused by prolonged CV treatment was not the explanatory mechanism. Studies that presented time on CV as a causal factor of worsening prognosis adjusted the effect for APACHE score and ventilatory settings but not for pH [17,18]. Only a retrospective study by Mehta and colleagues [14] mentioned time on CV as a predictor of mortality independent of age, APACHE II score and baseline pH. The strength of the effect and whether the association was weakened by the adjustment were not mentioned.

HFOV is a promising candidate for influencing mortality in ARDS patients. Research has demonstrated remarkable differences in mortality related to ventilation. These differences could be mainly attributed to ventilation strategies. There is now less discussion about the current optimal ventilation strategies in CV and HFOV [23]. The challenge seems to be to select the appropriate patients that benefit from HFOV compared with CV [16,24]. Predicting mortality has proven to be difficult because of the heterogeneous nature of ARDS. Yet ventilatory strategies have shown a constant treatment effect independent of predisposing clinical conditions [24]. In a recent publication of a randomized trial, it was hypothesized that the level of OI could determine which patients would receive a relative benefit from HFOV compared with CV [11]. This might oppose a more elective approach in which patients with ARDS are put on HFOV as quickly as possible to avoid prolonged ventilation on CV rather than waiting until a certain level of OI has been reached, as has been suggested [18]. However, the reviewed evidence presented in this report does not support that early HFOV in ARDS would be more beneficial, but that patients should be stratified by OI in future HFOV trials.

## Conclusion

Prolonged ventilation on CV prior to HFOV was not related to mortality. OI was associated with mortality independently of other disease markers and could be important for selecting ARDS patients that could benefit from HFOV.

## Key messages

• 	Prior time on CV, age, APACHE II score, OI and pH were associated with mortality in cohorts of patients treated with high frequency oscillatory ventilation.

• 	Prolonged ventilation on CV was not causally associated with higher mortality

• 	The OI may be a stratification factor to select patients that will benefit from HFOV in future trials

## Abbreviations

ARDS = acute respiratory distress syndrome; CI = confidence interval; CV = conventional mechanical ventilation; FiO_2 _= fraction of inspired oxygen; HFOV = high frequency oscillatory ventilation; MAP = mean airway pressure; OI = oxygenation index; PaCO_2 _= pressure of arterial carbon dioxide; PaO_2 _= arterial partial pressure of oxygen; RR = relative risk.

## Competing interests

The authors declare that they have no competing interests.

## Authors' contributions

CWB initiated the study. AJV and CSPMU participated in its design and helped to draft the manuscript. CWB and CSPMU performed the statistical analyses and wrote the manuscript. All authors read and approved the final manuscript.

## Figures and Tables

**Table 1:** Comparison of survivors and non-survivors treated with high frequency oscillatory ventilation

	Survival		OR (crude)
	No (60) Mean	Yes (33) Mean	

Age	51.2	42.3	1.14
APACHE II	26.1	22.4	1.12
TimeCV	6.2	4.0	1.38
pH	7.26	7.33	0.74^a^
PaCO_2_	54.6	43.8	1.07
PAF	91.8	94.8	0.90
OI	34.0	26.0	1.05
PIP	36.7	34.1	1.61
PEEP	14.5	13.9	1.09
MAP	24.0	22.9	1.81
FiO_2_	0.90	0.84	1.05^a^

Values are presented as pooled means of studies weighted by number of patients.

^a^OR per 0.01 unit change. FiO_2 _= fraction of inspired oxygen; MAP, mean airway pressure (cmH_2_O); OI, oxygenation index; OR, odds ratio; PaCO_2_, pressure of arterial carbon dioxide (mmHg); PAF, pressure of arterial oxygen (mmHg)/fraction of inspired oxygen; PEEP, peak end-expiratory pressure (cmH_2_O); PIP, peak inspiratory pressure (cmH_2_O); TimeCV, time on conventional mechanical ventilation (CV) prior to high frequency oscillatory ventilation (days).

**Table 2:** Linear regression analysis of determinants of mortality in high frequency oscillatory ventilation

Study	Mort	Vent	Surv	NoPat	Age	Sex	APACHE II	TimeCV	pH	PaCO_2_	PAF	OI	PIP	PEEP	MAP	FiO_2_
[15]	0.53			17	38.0	0.5	23.3	5.0		44.7		47.8	54.3	18.3		
[22]	0.20			5	36.6	2.6	28.7	6.0								
[18]	0.67		0.33	24	48.4	8.3	21.5	5.7		55.1	98.9	32.5	36.7	14.5	24.3	0.78
[13]	0.37	0.43	0.36	75	48.0	0.5	22.0	2.7	7.37	44.0	114.0	24.0	39.0	13.0	22.0	0.71
[21]	0.31			16	38.0		26.6	7.0	7.30	8.4		27.4	35.1	11.6		
[17]	0.43			42	49.0	0.7	28.0	3.0	7.33	57.0	94.0	23.0	35.0	15.0	24.0	1.00
[14]	0.62	0.53	0.18	154	47.9	56.6	24.0	5.7	7.28	53.2	91.2	31.4	36.1	14.0	24.1	0.86
[20]	0.32			25	42.4			4.9			97.0	26.8				
[11]	0.43	0.19	0.46	37	50.7	14.3	21.1	2.1	7.30	53.5		25.2	33.1	13.9	21.5	0.84

Values are presented as pooled means of studies. FiO_2_, fraction of inspired oxygen; MAP, mean airway pressure (cmH_2_O); Mort, mortality incidence at 30 days; NoPat, number of patients; OI, oxygenation index; PaCO_2_, pressure of arterial carbon dioxide (mmHg); PAF, pressure of arterial oxygen (mmHg)/fraction of inspired oxygen; PEEP, peak end-expiratory pressure (cmH_2_O); PIP, peak inspiratory pressure (cmH_2_O); Surv, survival at 30 days without ventilation; TimeCV, time on conventional mechanical ventilation (CV) prior to high frequency oscillatory ventilation (days); Vent, ventilation at 30 days.
